# The Effectiveness of Various Salacca Vinegars as Therapeutic Agent for Management of Hyperglycemia and Dyslipidemia on Diabetic Rats

**DOI:** 10.1155/2017/8742514

**Published:** 2017-02-14

**Authors:** Elok Zubaidah, Widya Dwi Rukmi Putri, Tiara Puspitasari, Umi Kalsum, Dianawati Dianawati

**Affiliations:** ^1^Department of Food Science and Technology, Brawijaya University, Malang 65145, Indonesia; ^2^Department of Pharmacology, Faculty of Health and Biomedicine, Brawijaya University, Malang 65145, Indonesia; ^3^Faculty of Food Science and Nutrition, Universiti Malaysia Sabah, Kota Kinabalu 88400, Malaysia

## Abstract

The aim of this study was to explore the potency of salacca vinegar made from various Indonesian salacca fruit extracts as therapeutic agent for hyperglycemia and dyslipidemia for STZ-induced diabetic rats. The rats were grouped into untreated rats, STZ-induced diabetic rats without treatment, and STZ-induced diabetic rats treated with Pondoh salacca vinegar, Swaru salacca vinegar, Gula Pasir salacca vinegar, Madu salacca vinegar, or Madura salacca vinegar. Parameter observed included blood glucose, total cholesterol (TC), high density lipoprotein (HDL), low density lipoprotein (LDL), triglyceride (TG), malondialdehyde (MDA), superoxide dismutase (SOD), and pancreas histopathology of the samples. The results demonstrated that all salacca vinegars were capable of reducing blood sugar (from 25.1 to 62%) and reducing LDL (from 9.5 to 14.8 mg/dL), TG (from 58.3 to 69.5 mg/dL), MDA (from 1.1 to 2.2 mg/dL), and TC (from 56.3 to 70.5 mg/dL) as well as increasing HDL blood sugar of STZ-induced diabetic Wistar rats (from 52.3 to 60 mg/dL). Various salacca vinegars were also capable of regenerating pancreatic cells. Nevertheless, the ability of Swaru salacca vinegar to manage hyperglycemia and dyslipidemia appeared to be superior to other salacca vinegars. Swaru salacca vinegar is a potential therapeutic agent to manage hyperglycemia and dyslipidemia of STZ-induced diabetic rats.

## 1. Introduction

Diabetes as one of metabolic diseases can be a major cause of complication such as “blindness, kidney failure, heart attack, and stroke” and leads to severe devastation on body's coordination such as nervous tension and blood vessels [1, 2]. In 2014, the number of people with diabetes reached 422 million. About 1.5 million deaths were because of diabetes and another 2.2 million deaths were likely due to high blood glucose [3]. Dietary, healthy lifestyle, and drug treatment are crucial to manage patients with diabetes. Vinegar has been used more than a decade to support optimally the diabetes management and to prevent diabetic complications [4]. Acetic acid is the main ingredient of vinegar with the concentration from 4 to 8%, whereas vitamins, mineral salts, amino acids, polyphenolic compounds, nonvolatile organic acids, and antioxidants may present in small amounts depending upon the sources [5]. Vinegar was effective in reducing blood sugar and regenerating the pancreas *β* cells of diabetic Wistar rats and was capable of decreasing LDL, triglycerides, and MDA as well as increasing HDL in their blood glucose [6, 7]. Consumption of vinegar by diabetic patients positively affected insulin activity resulting in a controllable blood sugar [6].

Many food resources have been studied as raw materials of vinegar, including apple, pineapple, unpolished and polished rice, and persimmon [5, 8, 9]. Apple vinegar and white rice vinegar were proven effective in lowering blood sugar level, improving pancreatic *β* cell function and lipid profile [5, 10]. Our previous study showed that a decrease in triglycerides and LDL along with an increase in HDL of diabetic rats treated with apple vinegar was due to the presence of acetic acid [11]. Acetic acid contained in vinegar plays a role in controlling the blood sugar level and also functions in reducing glycemic index of diabetic rats [5]. Antioxidants in apple vinegar could help in regenerating the deteriorated pancreas *β* cells resulting in an improvement of insulin secretion. Reducing triglycerides (TG) along with an increase in HDL was likely due to polyphenol effect of apple vinegar [12]. Polyphenols in apple were found to decrease LDL concentration of healthy subjects [13] and to increase HDL serum of experimental rats [14].

*Salacca zalacca*, a native Indonesian fruit which is also commonly known as snake fruit, has been proven as a potential raw material to produce high quality vinegar [11]. In vivo study carried out by [15] demonstrated that salacca has a bioactive characteristics and positively influenced lipid plasma profile and antioxidant activity on rat's plasma. Consumption of salacca fruit regularly was capable of reducing the risk of coroner atherosclerosis and blood coagulation [15]. Salacca is also known to have higher antioxidant content as compared to that in apples and lemons [16]. It contains fiber, vitamin A, vitamin B1, vitamin C, high carbohydrates content, and high antioxidant activity [1]. Zubaidah et al. [11] revealed that salacca vinegar prepared from Swaru salacca fruit was capable of decreasing LDL, triglycerides, total cholesterol, and increasing HDL serum plasma diabetic rats. Salacca vinegar made from salacca fruit extract indicated a superior functional capability to apple vinegar [17].

Various salacca fruits which spread in many areas in Indonesia have different characteristics, including the taste, flavor, and chemical compositions. Therefore, the difference in their efficacy as salacca vinegar in managing metabolic diseases could be predicted. In this study, we explored the potency of salacca vinegars made from various types of Indonesian salacca namely, Pondoh, Gula Pasir, Madura, Madu, and Swaru as a therapeutic agent for hyperglycemia and hyperlipidemia of STZ-induced diabetic rats. The capability of the various salacca vinegars in regenerating the pancreatic *β* cells of the diabetic rats was also ascertained.

## 2. Materials and Methods

### 2.1. Materials

Noncommercial salacca vinegars made from Pondoh, Gula Pasir, Madura, Madu, and Swaru salacca fruits were prepared according to the method of [12]. Chemical composition of salacca vinegar made from selected local salacca types can be seen in Table 1.

White female Wistar rats* Rattus norvegicus* (age 2.5–3.0 months; body weight 150–200 g) were obtained from laboratory of Pharmacology, Faculty of Health and Biomedicine, Brawijaya University, Malang. Comfeed PARS feed (PT. JapfaComfeed Indonesia Tbk.), miliQ water,* Streptozotocin* (STZ) (Nacalai Tesque, Kyoto, Japan), ether,* Buffer Neutral Formalin *(BNF) 10%, HCL 1 N, TCA, Na-Thiobarbiturate, PBS, xanthine, xanthine oxidase, NBT,* hematoxylin eosin* (HE), and paraffin were all obtained from Laboratory of Pharmacology and Laboratory of Pathology Anatomy, Faculty of Health and Biomedicine, Brawijaya University, Malang.

#### 2.1.1. Animals and Diet

The study was approved by Animal Ethical Committee of Brawijaya University, Malang. All the rats were adapted to the laboratory conditions for 7 days before further treatment. Rats were provided Comfeed PARS standard diet and drinking water ad libitum. Body weight of the experimental rats was determined at the end of the adaptation period to confirm whether they were in a healthy condition. The initial blood glucose level of the rats after being fasted for 8–10 hours was determined to ensure that no rat developed congenital diabetes. Once the adaptation period finished, the experiment started.

### 2.2. Experimental Design

Experimental design used was* True Experimental Design: Pre and Post Test with Control Group Design.* The experimental rats were totally 35 animals which were basically divided into two groups. Each group was subdivided into control (2 subgroups: positive and negative controls) and diabetic groups treated with different salacca vinegars (5 subgroups); hence, each group was consisted of 5 rats. All rats were housed in controlled conditions at room temperature of 23–25°C, 55–60% humidity, and a 12 h light/dark cycle.

The determination of 5 rats per group which were considered as a repetition was based on the reference of Yitnosumarto [18] with a statistical formulation as follows:

(1) $$\begin{array}{c} \left(\;t-1\;\right)\left(\;r-1\;\right)>15, \end{array}$$

where *t* is treatment and *r* is repetition.

Since we have 7 treatments, the number of animals per group is

(2) $$\begin{array}{c} \left(\;7-1\;\right)\left(\;r-1\;\right)>15; \end{array}$$

hence *r* = 5.16 or ≈5.

For STZ-induced diabetic rats, STZ was prepared freshly and was injected intraperitoneally at a concentration of 50 mg/kg of body weight using force feeding needle. After STZ injection, each rat was given a 5% glucose solution for 48 hour. Determination of blood glucose level of the diabetic rats was carried out after 3 days of STZ induction. Only the hyperglycemic rats with the blood glucose level of >126 mg/dL were chosen in this study. The calculation of day 0 was started at the 3rd day after STZ induction.

Details of each treatment can be seen as follows:

  Negative control (P0): normal diet for rats, without STZ induction.
  Positive control (P1): normal diet for rats, with STZ induction to create diabetic rats' model.
  Treatment 1 (P2): normal diet for rats induced with STZ and served with Pondoh salacca vinegar (0.4 mL/rat/day).
  Treatment 2 (P3): normal diet for rats induced with STZ and served with Swaru salacca vinegar (0.4 mL/rat/day).
  Treatment 3 (P4): normal diet for rats induced with STZ and served with Gula Pasir salacca vinegar (0.4 mL/rat/day).
  Treatment 4 (P5): normal diet for rats induced with STZ and served with Madu salacca vinegar (0.4 mL/rat/day).
  Treatment 5 (P6): normal diet for rats induced with STZ and served with Madura salacca vinegar (0.4 mL/rat/day).

### 2.3. Blood Glucose Level Determination

Before measuring the blood glucose, rats were fasted for 10–12 hours. The blood glucose level measurement was conducted on days 0, 7, 14, 21, and 28 using* glucose oxidase* biosensor method. The blood was taken from rats' tail point which was stabbed with syringe 1 mL (syringe* One Med *brand); then the blood was contacted with glucometer strip GlucoDr™ brand model AGM-2100 (produced by Allmedicus Co. Ltd., Korea).

### 2.4. Determination of Lipid Profile Levels, SOD, and MDA

Blood samples were collected from heart tissues at the end of the treatment period (day 28) for assays of lipid profiles, SOD (superoxide dismutase) activity, and MDA (malondialdehyde) concentration by dissecting the abdominal muscle. Levels of total cholesterol, HDL (high density lipoprotein), and LDL (low density lipoprotein) were analyzed by CHOD-PAP method, whereas triglyceride levels were analyzed by GPO-PAP method [19]. The SOD activity was assayed using the method of [20] at 480 nm for 4 min with a UV-Vis spectrophotometer (Hitachi U-2000). The activity was expressed as the amount of enzyme that inhibits the oxidation of epinephrine by 50%, which is equal to 1 U per milligram of protein. The level of lipid peroxidation was estimated based on the concentration of thiobarbituric acid-reactive MDA using the method of [21].

### 2.5. Pancreas Histopathology

At the end of 28-day experiment, the all experimental rats were euthanized with xylazine (5 mg/kg) and Ketamine HCl (40 mg/kg). Tissue sample of pancreas was collected and was fixed in 10% neutral buffered formalin, dehydrated, embedded in paraffin, sectioned at 3–5 *μ*m thickness, and stained with hematoxylin eosin staining for light microscopic evaluation (microscope Olympus CX21) [5]. The sections including pancreas tissue morphology and degradation of Langerhans islets were qualitatively observed.

## 3. Result

### 3.1. Blood Glucose Level

The blood glucose levels of treated rats during 4 weeks are presented in Table 2.

At week 0, the blood glucose levels of the controls and the STZ-induced rats treated with various salacca vinegars showed no significant difference. It indicated the uniformity of the blood glucose levels of the samples at the initial experiment. At week 4, however, a significant difference between the treatments was detected (*P* < 0.05). The blood glucose levels of STZ-induced diabetic rats treated with various salacca vinegars were significantly lower (*P* < 0.05) than that of untreated diabetic rats. The highest decrease in blood glucose level of diabetic rats was 61.7% when they were treated with Swaru salacca vinegar (P3); followed by 58.8% due to Madura salacca vinegar treatment (P6).

### 3.2. Pancreas Histopathology

**Figure 1:**
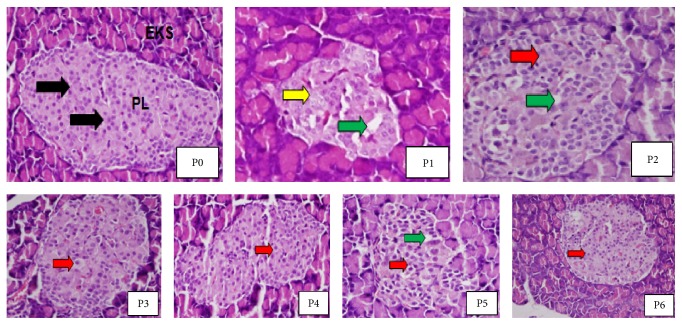
Histopathology of Langerhans islets of *β* pancreas cells (400x magnifications). P0 = normal rats without STZ induction; P1 = STZ-induced diabetic rats with no salacca vinegar treatment; P3 = STZ-induced diabetic rats treated with Swaru salacca vinegar; P4 = STZ-induced diabetic rats treated with Gula Pasir salacca vinegar; P5 = STZ-induced diabetic rats treated with Madu salacca vinegar; and P6 = STZ-induced diabetic rats treated with Madura salacca vinegar. PL = Langerhans islets, EKS = exocrine glands, black arrow = normal cells, green arrow = empty room due to necrosis, yellow arrow = loss of nucleus, and red arrow = endocrine cells which started doing regeneration toward their normal configuration.

Cell histopathology of Langerhans islets of the two controls and the diabetic rats treated with salacca vinegars after 1 month treatment was shown in Figure 1.

In the diabetic rats with no treatment (Figure 1; P1), degranulation in the cytoplasm of the deteriorating cells occurred along with necrosis and hydropic degeneration; shrunken Langerhans islets were noticeable. On the contrary, the diabetic rats treated with different types of salacca vinegars (P2 to P6) started regeneration of endocrine cells toward normal indicated by red arrows. Nevertheless, necrotic cells of diabetic rats treated with Pondoh salacca vinegar (P2) and Madu salacca vinegar (P5) still appeared together with light hydropic degeneration and degranulation. The most obvious regeneration of the deteriorated *β* pancreas cells of STZ-induced diabetic rats was indicated by Swaru salacca vinegar (P3) followed by Madura salacca vinegar (P6) treatments.

### 3.3. SOD and MDA Levels

Levels of SOD and MDA serum of STZ-induced diabetic rats are shown in Table 3.

The SOD levels of diabetic rats treated with Pondoh, Swaru, and Madura salacca vinegars (P2, P3, and P6, resp.) were significantly higher (*P* < 0.05) than that of positive control (P1). In the line with SOD results, all MDA levels of diabetic rats treated with various salacca vinegars were lower than that of positive control (P1). The highest SOD level along with the lowest of MDA level, which was 56.99 U/mL and 1.08 mg/mL, respectively, was demonstrated by the diabetic rats treated with Swaru salacca vinegar (P3), followed by those treated with Madura salacca vinegar (P6), which was 48.27 U/mL and 1.37 mg/mL, respectively.

### 3.4. Lipid Profile

The levels of HDL, LDL, and total cholesterol of diabetic rats treated with Pondoh, Swaru, Gula Pasir, and Madura salacca vinegars (P2, P3, P4, and P6, resp.) were significantly different (*P* < 0.05) as compared to those of positive control (P1) (Table 4).

Similarly, TG levels of all diabetic rats treated with various salacca vinegars were significantly lower (*P* < 0.05) than that of positive control (P1). The best lipid profile was demonstrated by diabetic rats treated with Swaru salacca vinegar (P3) and Madura salacca vinegar (P6), in which the levels of LDL, TG, and total cholesterol were not significantly different as compared to that of negative control (P0).

## 4. Discussion

In current study, the daily consumption of salacca vinegar from diverse salacca fruits by STZ-induced diabetic rats indicated their capability as a therapeutic agent for hyperglycemia and hyperlipidemia as demonstrated by blood glucose level, islet *β* cell performance, SOD, and MDA levels, as well as HDL, LDL, TG, and total cholesterol levels. The effectiveness of salacca vinegars in lowering blood glucose level was confirmed with the finding of [12]. The result was also in agreement with that of [22] who demonstrated a reducing effect of apple cider vinegar on blood glucose levels in diabetic rats. Similarly, [10] found a decrease in HbA1-c of diabetic rats treated with apple cider vinegar. Acetic acid, the main compound of vinegar, appeared to play its important role in blood glucose level reduction [23]. Some mechanisms related to how vinegar reduces blood glucose concentrations have been proposed. It might be through an inhibition of acetic acid on the activity of disaccharide enzymes such as sucrase, maltase, trehalase, and lactase in small intestine [24], as well as amylases [22, 25]. Ogawa et al. [24] stated that acetic acid in vinegar may restrict the digestion of starch resulting in a decreased amount of glucose absorbed into the blood stream after meal time. O'Keefe et al. [26] revealed the ability of vinegar to reduce the rate of gastric emptying causing the delay of carbohydrates absorption and satiety. Acetic acid may also maintain blood glucose concentration by improving an uptake of glucose from the blood stream [27].

An improved performance of endocrine cells of the STZ-induced diabetic rats treated with different types of salacca vinegars (Figure 1) showed the capability of the vinegars to regenerate toward the normal cells. The result was in agreement with that of [5]. They found that the islet *β* cell amount of STZ-induced diabetic rats was considerably higher when treated with white rice vinegar for a month than that of untreated group; islet area of the treated diabetic rats also increased. This implied that salacca vinegar might somewhat protect *β* cells from the toxicity effect of STZ so that the partial cells were able to secrete insulin, which is in agreement with the finding of [28]. The improvement of *β* cell treated with salacca vinegars could be the reason why a decrease in blood glucose level occurred. Improved *β* cells result in an increase in insulin secretion. Insulin inhibits lipolysis, gluconeogenesis, and glycogenolysis that results in a decrease in free radicals and ROS [29].

A decrease in blood glucose level along with an improvement of *β*-pancreas cells appears to be correlated with total phenol and antioxidant activity of salacca vinegars made from various salacca fruits. Pearson correlation analyzed from Table 1 demonstrated a strong positive correlation between total phenol and antioxidant activity (*r* = 0.91; *P* < 0.05); a strong negative correlation between blood sugar level and total phenol (*r* = −0.99; *P* < 0.05); and a strong negative correlation between blood sugar level and antioxidant activity (*r* = −0.95; *P* < 0.05). Flavonoids and tannins, which are categorized as group of phenols, are antioxidants that may play their roles in pancreas *β* cells' regeneration. In people with hyperglycemia, *β* cell role is gradually deviated; glucose-induced insulin secretion becomes damaged along with *β* cells' degranulation; a reduction of *β* cells quantity sometimes also occurs. The antioxidant treatment suppressed apoptosis in *β* cells without changing the rate of *β* cell proliferation, supporting the hypothesis that, in chronic hyperglycemia, apoptosis induced by oxidative stress causes reduction of *β* cell mass [30]. To date, phenolic compounds are used as one indicator to ascertain the quality and the originality of vinegar [31]. Since total phenolic compounds and antioxidant activity of Swaru salacca vinegar were 233.0 (mg/L GAE) and 68.1%, respectively, this vinegar appeared more effective in improving Langerhans of pancreas islet as compared to other salacca vinegars (Figure 1; P3).

Decrease in MDA levels along with increase in SOD levels of blood serum of STZ-induced rats treated with various types of salacca vinegar (Table 3) indicated an improvement of pancreas *β* cells. This result is in agreement with that of [32]. Overexpression of catalase and superoxide dismutase (SOD) has been shown to protect human islets [33, 34] and *β* cell lines [35, 36] against oxidative stress. The precise mechanism on how SOD level increases due to salacca vinegar treatment is still unclear, but it might be due to the presence of flavonoids. Flavonoids are capable of increasing the activity of* nuclear factor erythroid 2-related factor 2* (Nrf2) that functions to synthesize endogen antioxidants such as superoxide dismutase (SOD) enzyme [21]. Correspondingly, flavonoids may ameliorate blood lipids and blood glucose levels of STZ-induced diabetic rats by increasing SOD activity and glucose transporter 4 (GLUT-4) expression, as well as reducing MDA level and CYP2E1 expression [37].

In corroboration with SOD and MDA levels, the lipid profile of diabetic rats treated with all salacca vinegars also improved as being indicated by high level of HDL along with low level of LDL, TG, and TC (Table 4). Similarly, Moon et al. [38] reported that persimmon-vinegar decreased serum TC concentration in mice. Fushimi et al. [39] also reported a decrease in serum TC when 0.3% (w/w) dietary acetic acid was managed for 19 days of regular diet added with 1% cholesterol. Apple cider vinegar was also effective in decreasing serum LDL and TG and increasing serum HDL of normal and diabetic rats [10].

## 5. Conclusion

The diabetic rats treated with Swaru salacca vinegar (P3) showed a significant decrease in blood glucose level (61.69%) and an improvement of lipid profile indicated by low LDL level (9.5 mg/dl), low TG level (58.25 mg/dl), high HDL level (60 mg/dl), low total cholesterol (56.25 mg/dl), and low MDA level (1.076 mg/dl) as well as low SOD level (56.986 U/ml) which are comparable to negative control (healthy rats). The histopathology observation of diabetic rats treated with salacca vinegar made from various types of salacca fruits indicated an improvement on pancreas cells. Overall, salacca vinegars particularly the one which was prepared from Swaru salacca fruit can be a potential therapy agent to manage hyperglycemia and dyslipidemia of diabetic rats. However, particular observation on the bioflavonoid compounds and tannin in salacca vinegar and how their roles in managing hyperglycemia and dyslipidemia need to be carried out for future study.

## Figures and Tables

**Table 1:** Chemical compounds of various salacca vinegars.

Salacca variety	Total acid content (%)	pH	Total phenolic content (mg/L GAE)	Antioxidant activity (%)	Sugar content (°brix)	Alcohol content (%)
Pondoh	1.421	2.713	195.167	36.707	0.048	0
Swaru	1.046	2.937	233.000	68.113	0.035	0
Gula Pasir	1.463	2.713	142.500	34.643	0.039	0
Madu	1.236	2.813	111.000	23.527	0.036	0
Madura	1.074	2.893	213.167	59.520	0.033	0

**Table 2:** Blood glucose levels of diabetic rats treated with or without various salacca vinegars during 4 weeks.

Treatment	Blood glucose level (mg/dL)		Reduction (%)
	Week 0	Week 4	
P0	98.6 ± 11.33^b^	107.2 ± 2.39^e^	8.519
P1	385.0 ± 37.60^a^	510.8 ± 63.28^a^	32.662
P2	319.1 ± 62.79^a^	178.8 ± 7.32^cd^	−43.966
P3	298.3 ± 18.57^a^	114.3 ± 12.45^de^	−61.693
P4	316.3 ± 7.97^a^	224 ± 18.85^bc^	−29.169
P5	369.1 ± 34.38^a^	276.3 ± 42.11^b^	−25.135
P6	353.0 ± 27.98^a^	145.4 ± 32.87^de^	−58.810

Different letters in each column showed significant differences (*P* < 0.05).

Values are presented as means of four repetitions ± SD.

**Table 3:** Level of SOD and MDA of blood serum of diabetic rats treated with or without various salacca vinegars.

Treatments	SOD levels (U/ml)	MDA levels (mg/dL)
P0	59.701 ± 4.77^a^	0.780 ± 0.15^e^
P1	28.246 ± 12.74^c^	3.133 ± 0.24^a^
P2	43.182 ± 2.35^b^	1.533 ± 0.11^c^
P3	56.986 ± 1.29^a^	1.076 ± 0.21^de^
P4	40.723 ± 4.56^bc^	1.735 ± 0.08^c^
P5	38.759 ± 5.84^bc^	2.227 ± 0.36^b^
P6	48.265 ± 2.49^ab^	1.365 ± 0.09^cd^

Different letters in each column showed significant differences (*P* < 0.05).

Values are presented as means of four repetitions ± SD.

**Table 4:** Lipid profile of diabetic rats treated with or without various salacca vinegars.

Treatment	HDL levels (mg/dL)	LDL levels (mg/dL)	Triglyceride levels (mg/dL)	Total cholesterol levels (mg/dL)
P0	68.00 ± 2.24^a^	8.80 ± 1.92^c^	45.41 ± 6.23^c^	49.80 ± 4.82^e^
P1	40.75 ± 4.79^e^	19.25 ± 3.59^a^	91.75 ± 6.50^a^	75.50 ± 5.45^a^
P2	52.25 ± 3.10^cd^	11.50 ± 2.38^bc^	64.50 ± 3.42^b^	63.25 ± 3.30^bc^
P3	60.00 ± 3.65^b^	9.50 ± 1.29^c^	58.25 ± 4.65^bc^	56.25 ± 4.79^de^
P4	50.25 ± 2.50^cd^	12.25 ± 2.22^bc^	66.25 ± 4.99^b^	65.75 ± 5.12^bc^
P5	47.75 ± 2.99^de^	14.75 ± 1.71^ab^	69.50 ± 3.70^b^	70.50 ± 5.97^ab^
P6	56.4 ± 3.58^bc^	10.40 ± 2.07^bc^	61.60 ± 4.22^b^	60.20 ± 4.32^cd^

Different letters in each column showed significant differences (*P* < 0.05).

Values are presented as means of four repetitions ± SD.
